# Short-term outcome of diverting loop ileostomy reversals performed by residents: a retrospective cohort prognostic factor study

**DOI:** 10.1007/s00384-023-04390-0

**Published:** 2023-04-21

**Authors:** Clara von Savigny, Mazen A. Juratli, Christine Koch, Tatjana Gruber-Rouh, Wolf O. Bechstein, Teresa Schreckenbach

**Affiliations:** 1 Department of General, Visceral, Transplantation, and Thoracic Surgery, Goethe University Frankfurt/Main, Frankfurt University Hospital and Clinics, Theodor-Stern-Kai 7, 60596 Frankfurt/Main, Germany; 2 Department of General, Visceral and Transplant Surgery, Muenster University Hospital, Muenster, Germany; 3 Department of Internal Medicine, Goethe University Frankfurt/Main, Frankfurt University Hospital and Clinics, Theodor-Stern-Kai 7, 60596 Frankfurt/Main, Germany; 4 Institute of Diagnostical and Interventional Radiology, Goethe University Frankfurt/Main, Frankfurt University Hospital and Clinics, Theodor-Stern-Kai 7, 60596 Frankfurt/Main, Germany

**Keywords:** Resident involvement, Surgical trainee, Education, Diverting loop ileostomy, Patient care

## Abstract

**Aim:**

The reversal of diverting loop ileostomy (DLI) is one of surgical trainees’ first procedures. Complications of DLI reversal can cause life-threatening complications and increase patient morbidity. This study compared DLI reversals performed by surgical trainees with those by attending surgeons.

**Method:**

This retrospective cohort study was performed at a single primary care center on 300 patients undergoing DLI reversal. The primary outcome was morbidity, according to the Clavien-Dindo classification (CDC), with special attention paid to the surgeon’s level of training. The secondary endpoint was postoperative intestinal motility dysfunction.

**Results:**

Surgical trainees had significantly longer operation times (*p* < 0.001) than attending surgeons. Univariate analyses revealed no influence on the level of training for postoperative morbidity. First bowel movement later than 3 days after surgery was a significant risk factor for CDC $\ge$ 3 (OR, 4.348; 96% CI, 1670–11.321; *p* = 0.003). Independent risk factors for surgical site infections (SSIs) were an elevated BMI (OR, 1.162; 95% CI, 1.043–1.1294; *p* = 0.007) and a delayed bowel movement (OR, 3.973; 95% CI, 1.300–12.138; *p* = 0.015). For postoperative intestinal motility dysfunction, an independent risk factor was a primary malignant disease (OR, 1.980; 95% CI, 1.120–3.500; *p* = 0.019), and side-to-side stapled anastomosis was a protective factor (OR, 0.337; 95% CI 0.155–0.733; *p* = 0.006).

**Conclusion:**

Even though surgical trainees needed significantly more time to perform the surgery, the level of surgical training was not a risk factor for increased postoperative morbidity. Instead, delayed first bowel movement was predictive of SSI.

**Supplementary Information:**

The online version contains supplementary material available at 10.1007/s00384-023-04390-0.

## Introduction

Maximum patient safety, quality management, efficiency, and cost-effectiveness have become increasingly important during the last decades. Especially for surgical procedures, high levels of performance are required. As such, a well-founded surgical training system for surgical residents is necessary. However, patients’ willingness to consent to resident participation is low, with only 14% consenting to resident operating and staff surgeon observing, while at least 56% would consent to a resident assisting staff during a procedure [1, 2].

At the same time, residents’ participation in surgeries and procedures is essential for surgical education and for ensuring the demanded good-quality medical care in the future. Analyses of comparative outcomes of resident versus attending surgeons performing surgery revealed that resident-performed surgery takes longer, causes more Clavien-Dindo classification (CDC) grade I and IIIa complications, and results in fewer deaths, which leads to the conclusion that resident-performed surgeries are safe in carefully selected patients [2]. However, because of the heterogeneity present in the outcomes, generalizability should be prevented [2, 3]. Diverting loop ileostomy (DLI) reversal is a reasonable surgery for beginners as it is a simple procedure and helpful toward learning the proper construction of bowel anastomosis. DLIs are often temporarily created to prevent the life-threatening complications associated with rectal surgery. DLI can decrease the rate of symptomatic anastomotic leakage and the need for emergency reoperation [4].

However, DLI reversal itself is an operation with its own possible complications, such as small bowel obstruction, anastomotic leakage, wound infection, and incisional hernia [5, 6]. A systematic review of 48 studies, including 6107 patients, showed an overall morbidity rate of 17.3% and a mortality rate of 0.4% [5]. These results are supported by Hussain et al., who show that more complications were reported by patients with a delayed DLI and those who underwent small bowel resection but not when a surgical trainee performed the operation [6]. In contrast, another study reported that the main factor significantly affecting postoperative morbidity was the surgeon’s experience [7].

The aim of this retrospective study is to compare the outcome after DLI reversals and identify risk factors for elevated morbidity with special attention to the surgeon’s level of training.

## Methods

This study is a retrospective cohort study performed at a single primary care center. The study was conducted in accordance with the Declaration of Helsinki and was approved by the Ethics Committee of Goethe University, Frankfurt, Germany (no. 435/14).The data used in this study were anonymized before use.

### Patients’ demographics and clinical data

All DLI reversals performed between January 2009 and December 2019 in the Department of the General and Visceral Surgery of the University Hospital Frankfurt/Main were included in this retrospective study. The exclusion criteria were combined surgery and emergency surgery.

Patient characteristics were collected from the electronic health records database (ORBIS, Agfa) and a dedicated University Cancer Center (UCT) database. We used the International Classification of Disease (ICD), 10th revision, German modification, and the Operation and Procedure Classification System (OPS) to identify eligible patients who met the inclusion criteria. The data were collected by one member of the research group and double-checked by one of the senior authors.

Patient information and tumor characteristics were obtained from the database, including sex, age, body mass index (BMI in kg/m^2^), physical status classification according to the American Society of Anesthesiologists (ASA), and primary disease leading to the loop ileostomy. Comorbidities included coronary artery disease, chronic obstructive pulmonary disease (COPD), chronic kidney disease (CKD), and diabetes mellitus. Risk factors, including alcohol and nicotine use, were also recorded.

The primary diseases leading to DLI were further categorized as either benign or malignant. Benign illnesses were ulcerative colitis, Crohn’s disease, sigmoid diverticulitis, perforation or ischemia of the intestine, rectal prolapse, fistula, and familial adenomatous polyposis (FAP). Malignant illnesses included carcinomas of the colon, rectum, and urogenital system.

### Surgical procedures and postoperative complications

All operation notes were closely scrutinized. The anastomosis types were end-to-end hand-stitched or side-to-side staple sutured, with or without resection of the small bowel. The resulting wound was closed using either the primary linear closure (PLC) or purse-string closure (PSC) technique.

Postoperative complications included surgical site infections (SSI), fascial dehiscence, intestinal motility dysfunction (e.g., diarrhea, vomiting, or more than 3 days to first bowel movement), and re-laparotomy. Postoperative complications were reviewed and graded using the Clavien-Dindo classification system [3]. Minor complications were categorized as either grade 1 or 2, while major complications were categorized as grade 3 or higher.

MTL30 as marker for surgical quality was calculated for every patient. It was rated positive, if a patient died within 30 days (M +), stayed in inpatient care more than 30 days (L +), and/or was transmitted to another acute care hospital (T +) [8, 9].

### Surgeons

Two groups of surgeons were defined. The first one included all surgical trainees independent of their year of training. The second group consisted of all attending surgeons.

### Study endpoints

The primary endpoint of the study was overall morbidity, which depended on the level of education of the surgeon. The secondary endpoints of the study were SSIs and intestinal motility dysfunctions.

### Outcome parameters

The following parameters were used to compare the outcome of DLI reversal between the two surgeon groups: postoperative complication grade 3 or higher classified according to the CDC classification, SSIs, and intestinal motility dysfunction.

### Statistics

All statistical analyses were performed using the Statistical Package for the Social Sciences for Windows (version 25.0; International Business Machines Corporation, Chicago, IL). Categorical variables are described in frequencies and percentages. They were compared using the chi-squared $\left({\chi }^{2}\right)$ test with Bonferroni correction or Fisher’s exact test, as appropriate. First, the ${\chi }^{2}$ test was performed on both groups of surgeons.

Continuous variables are represented as means with their standard deviation (SD) and range. They were compared using a one-way analysis of variance (ANOVA).

Logistic regression analysis was used in the Supplementary to identify factors associated with postoperative complications above or equal to CDC grade 3, SSIs, and intestinal motility dysfunction. The following parameters were included in the univariable logistic regression analysis: sex, age, BMI, ASA group, cardiovascular system, diabetes, primary disease, duration of surgery, anastomotic type, wound closure, level of training, time to reversal, delay to reversal, and days until first bowel movement. Variables with a *p*-value of < 0.05 in univariable logistic regression analyses were used for multivariable analyses. Results were expressed as an odds ratio (OR) with a 95% confidence interval (CI). A *p*-value of < 0.05 was considered statistically significant in all tests.

## Results

Between January 2009 and December 2019, 331 ileostomy reversals were performed. After excluding combination operations (*n* = 29) and emergency operations (n = 2), 300 cases were analyzed.

### Patients’ characteristics

The mean age was 60 years (SD, $\pm$ 14). One hundred eighty-five patients (61.7%) were male, and 115 patients (38.3%) were female. Two hundred five patients (68.3%) had received their loop ileostomy due to malignant diseases. No significant difference was found between the two surgeon groups based on the primary disease (*p* = 0.239). There was also no significant difference in the ages between the two groups (*p* = 0.362). However, sex differed significantly between the two groups (*p* = 0.009).

Two hundred eighty-seven patients (95.7%) had comorbidities, and these were equally distributed between the surgeon groups (*p* = 0.094) except for Crohn’s disease, which occurred significantly more often in the group of surgical trainees (5.9% vs. 1.6%, *p* = 0.047). The risk factors showed no significant difference (*p* = 0.76 (alcohol); *p* = 0.252 (nicotine)) as well as the ASA group (*p* = 0.88) (Table 1).

**Table 1:** Patients’ characteristics. The chi-square test was used to analyze the categorical variables. Pairwise comparisons between groups were made with Bonferroni correction. For continuous variables, one-way ANOVA analysis with Bonferroni correction was performed

	**All (%) (*n* = 300)**	**Surgical trainee (%) (*n* = 118)**	**Attending surgeon (%) (*n* = 182)**	***p*-value**
Sex (patient)				0.009
Male	185 (61.70)	62 (52.50)	123 (67.60)	
Primary disease				0.239
Benign	95 (31.70)	42 (35.60)	53 (29.10)	
Malign	205 (68.30)	76 (64.40)	129 (70.90)	
Comorbidities				
Cardiovascular disease	140 (46.70)	54 (45.80)	86 (47.30)	0.800
Diabetes mellitus	45 (15.00)	13 (11.00)	32 (17.60)	0.120
COPD	12 (4.00)	5 (4.20)	7 (3.80)	1.000
Chronic kidney failure	32 (10.70)	11 (3.70)	21 (11.50)	0.544
Liver disease	20 (6.70)	7 (5.90)	13 (7.10)	0.681
Crohn’s disease	10 (3.30)	7 (5.90)	3 (1.60)	0.047
Risk factors				
Nicotine	36 (12.00)	15 (12.70)	21 (11.50)	0.760
Alcohol	7 (2.30)	1 (0.80)	6 (3.30)	0.252*
ASA score (*n* = 119)				0.878
ASA < 3	53 (44.50)	20 (45.50)	33 (44.00)	
ASA $\ge$ 3	66 (55.50)	24 (54.50)	42 (56.00)	
	**All (*n* = 300)**	**Surgical trainee (*n* = 118)**	**Attending surgeon (*n* = 182)**	***p*-value**
Age in years (mean, SD, range)	60.36 (14.38) (21–90)	59.42 (14.58) (21–88)	60.97 (14.26) (21–90)	0.362
BMI (kg/m^2^) (mean, SD, range)	24.22 (4.67) (13–43)	24.37 (4.69) (13–42)	24.12 (4.68) (15–43)	0.645

*Fisher's exact test was used

### Surgical details

Two hundred sixty-eight patients (89.3%) received a hand-stitched anastomotic suture. More attending surgeons performed a stapled suture, which was statistically significant (*p* = 0.012). Regarding the wound closure technique, no difference was found between the two groups.

Complications classified according to the CDC classification (without CDC V) showed no significant difference between the groups (*p* = 0.211). Two patients (0.7%) in the attending surgeon group died (CDC V) following the ileostomy reversal from myocardial infarction (*n* = 1) and ventricular fibrillation (*n* = 1). The difference was not statistically significant (*p* = 0.521). Four patients (3.4%) in the surgical trainee group and 13 patients (7.1%) in the attending surgeon group underwent reoperation (*p* = 0.207). The reasons were anastomotic dehiscence (*n* = 9), adhesion ileus (*n* = 3), and other reasons (*n* = 5).

There was a significant difference between the groups regarding the duration of surgery (*p* < 0.001). The duration of surgery for residents was 85 min (SD, ± 27 min) and for attending surgeons, 59 min (SD, ± 25 min). No other differences were observed. For more details, see Table 2.

**Table 2:** Surgical details. The chi-square test was used to analyze the categorical variables. If appropriate, Fisher’s exact test was used (marked with *). Pairwise comparisons between groups were made with the Bonferroni correction. For continuous variables, one-way ANOVA analysis with Bonferroni correction was performed

	**Total (%) (*n* = 300)**	**Surgical trainee (%) (*n* = 118)**	**Attending surgeon (%) (*n* = 182)**	***p*-value**
Type of suture				**0.012**
End-to-end hand-stitched	268 (89.30)	112 (94.90)	156 (85.70)	
Side-to-side stapled suture	32 (10.70)	6 (5.10)	26 (14.30)	
Wound closure (*n* = 299)				0.891
PLC	93 (31.10)	35 (29.70)	58 (32.00)	
PSC	206 (68.90)	83 (70.30)	123 (68.00)	
Postoperative complications				
SSIs	19 (6.30)	9 (7.60)	10 (5.50)	0.459
Fascial dehiscence	2 (0.70)	1 (0.80)	1 (0.50)	0.757
Postoperative diarrhea	139 (46.30)	52 (44.10)	87 (47.80)	0.526
Postoperative paralysis	74 (24.70)	23 (19.50)	51 (280)	0.094
Postoperative vomiting	63 (21.00)	21 (17.80)	42 (23.10)	0.273
Bowel motility treatment	187 (62.30)	64 (54.20)	123 (67.60)	0.020
Re-laparotomy	17 (5.70)	4 (3.40)	13 (7.10)	0.170
Anastomotic dehiscence	12 (4.00)	3 (2.50)	9 (4.90)	0.377*
Clavien-Dindo Score (without CDC V)				0.211
No complications	92 (30.90)	45 (38.10)	47 (26.10)	
CDC I	149 (50.00)	53 (44.90)	96 (53.30)	
CDC II	36 (12.10)	14 (11.90)	22 (12.20)	
CDC III	19 (6.40)	5 (4.20)	14 (7.80)	
CDC IV	2 (0.70)	1 (0.80)	1 (0.60)	
Postoperative death (CDC V)	2 (0.70)	0	2 (1.1)	0.521*
MTL30				0.745*
Positive	10 (3.30)	3 (2.5)	7 (3.8)	
	**Total (*n* = 300)**	**Surgical trainee (*n* = 118)**	**Attending surgeon (*n* = 182)**	***p*-value**
Duration of surgery in minutes (mean, SD, range)	69.26 (28.70) (23–282)	85.08 (27.46) (32–282)	58.99 (24.58) (23–183)	** < 0.001**
Time to reversal of DLI in days (mean, SD, range)	151.73 (132.94) (16–946)	153.67 (127.46) (19–917)	150.47 (136.70) (16–946)	0.839
Time until first postoperative bowel movement in days (mean, SD, range)	4.26 (23.46) (0–12)	2.49 (1.11) (1–5)	5.5 (30.52) (0–12)	0.322
Length of hospital stay in days (mean, SD, range)	8.82 (9.81) (2–118)	8.4 (7.66) (3–63)	9.1 (11.00) (2–118)	0.550
Follow-up in months (mean, SD, range)	48.76 (41.08) (0–148)	49.75 (41.12) (0–141)	48.13 (41.15) (0–148)	0.739

### Logistic regression analysis

Based on univariable logistic regression analysis, the first bowel movement occurring more than 3 days after the operation correlated significantly to complications graded CDC = 3 (OR, 4.35; % CI, 1.67–11.32; *p* = 0.003) (Supplementary 1).

Risk factors for the postoperative development of SSIs in the multivariate logistic regression analysis were BMI (OR, 1.2; 95% CI, 1.0–1.3; *p* = 0.007) and days until first bowel movement (OR, 4.0; 95% CI, 1.3–12.1; *p* = 0.015) (Supplementary 2).

Multivariate logistic analysis for intestinal motility dysfunction revealed that having a primary malignant disease (OR, 2.0; 95% CI, 1.1–3.5; *p* = 0.019) was a risk factor. A stapled suture closure was a protective factor (OR, 0.3; 95% CI, 0.2–0.7; *p* = 0.006) (Supplementary 3).

## Discussion

This retrospective study shows that the level of training of the surgeon does not have a significant impact on the outcome of loop ileostomy reversals. No significant difference occurred in terms of postoperative complications, including wound infection, anastomotic leakage, diarrhea, vomiting, or postoperative paralysis. Postoperative complications classified according to the CDC showed no significant differences between the two groups of surgeons as well.

Nonetheless, by an average of about 25 min, surgical trainees needed significantly more time to complete the operation. Prolonged operative time is a risk factor for an increased likelihood of complications, including SSIs, postoperative bowel obstruction, bleeding, and intraabdominal abscess. One study described a 14% increase for every additional 30 min of operative time [10]. Another one observed a positive linear relationship between the risk for SSI and operative time, in which the operative time for patients with SSIs was 30 min longer on average [11]. Another study described an average 105 $\pm$ 29.6 min longer operative time as a risk factor for reoperation after loop ileostomy reversal [12]. The interpretation of the present retrospective study, however, does not support these findings as the duration of surgery was not associated with a higher risk for CDC $\ge$ 3, wound infection, or intestinal motility dysfunction. A possible explanation could be that the operation was almost always shorter than 2 h, the operative time threshold for a doubled likelihood of complications [10]. Furthermore, this was shorter than the previously described 105 $\pm$ 29.6 min, and the residents needed an average of 25 min more than the consultants. In addition, the level of training of the surgeon did not significantly correlate with CDC $\ge$ 3, wound infection, or intestinal motility dysfunction.

The skin closure technique played an important role in developing SSIs in ostomy reversal. Several studies describe significant differences between PSC and PLC techniques [13]. SSIs occurred significantly more often in the PLC group; PSC led to an 80% reduction in SSI compared with PLC. Furthermore, the wound closure technique was the only significant risk factor for SSIs, while surgery performed by a resident under supervision was not [1, 14, 15]. Even though not significant, the interpretation of this retrospective study shows an OR of 1.3 for interrupted sutures or skin staples compared with the purse-string suture. Both groups performed purse-string sutures more often by far (*n* = 206 (68.9%)).

Several risk factors for SSIs were detected. Diabetes mellitus might be an independent risk factor for SSIs [16] in addition to postoperative hyperglycemia as a risk factor for wound infections [17]. Patients with SSIs presented consistently higher blood glucose levels than those without [16]. The data collected in this study does not support establishing any significant correlation or causality relationships.

Another important risk factor for SSIs is obesity [18]. A BMI > 25 kg/m^2^ was associated with a higher risk for SSIs [19], which is supported by findings of a higher risk for SSIs among obese patients by body fat and increased risk with increasing body fat [19]. A BMI $\ge$ 30 kg/m^2^ showed a 1.5- to 1.6-fold higher risk for SSIs compared with a BMI < 30 kg/m^2^, and a BMI between 25 and 29.9 kg/m^2^ showed a 1.2-fold higher risk compared with normal-weight patients [19]. These findings are supported by another study in which obese patients (> 25% body fat in men, > 31% body fat in women) had a fivefold higher risk for SSIs than nonobese patients [17] and that risk increased as body fat increased.

In summary, there are multiple risk factors for SSIs, such as reduced fitness and patient frailty (e.g., elevated BMI and body fat, smoking, diabetes mellitus, and thicker subcutaneous fat), surgery duration and complexity, and most importantly, wound closure technique (primary linear skin closure), but not the level of training of the surgeon [20, 21].

Intestinal motility dysfunction, defined as postoperative diarrhea, vomiting, and more than three days until the first bowel movement, is an important marker for the success of abdominal surgery as it indicates whether bowel function has returned correctly. One significant protective factor for the development of intestinal motility dysfunction was the use of stapled anastomosis (OR, 0.38). Other studies support the present finding by showing a significantly reduced rate of postoperative bowel obstruction as well as shorter operative times [22–25]. As significantly more attending surgeons performed the stapled anastomosis (*n* = 26, 8.7% vs. *n* = 6, 5.1%; *p* = 0.012), while surgical trainees more often use hand-sewn approaches when operating on intestinal anastomoses, which could be another explanation for the attending surgeons’ shorter operative times. The level of training, however, had no significant influence on intestinal motility dysfunction.

Enhanced recovery after surgery (ERAS) is a concept developed to improve patients’ outcomes after surgery by following certain guidelines, including pre-, intra-, and postoperative interventions such as preoperative malnutrition reduction, no prolonged fasting, fluid and carbohydrate loading, short-acting anesthetic agents, avoidance of salt and liquid overload, prevention of nausea and vomiting, non-opioid oral analgesia, and early oral good intake, which is another important factor influencing the return of proper bowel function.

Eating earlier after surgery may reduce the length of the postoperative hospital stay and is likely to stimulate gut motility and, therefore, lead to an earlier return of bowel function [26, 27]. It does not make any significant difference regarding nutrient composition except for coffee consumption, which significantly reduced the time to first flatus and time to tolerance of solid foods as well as time to defecation and time to first bowel movement while being well-tolerated and low in cost [28, 29]. Similar results were generated here in the present study. Time from ileostomy reversal to the first day of regular enteral nutrition as well as time from the first day of regular enteral nutrition to the first day of defecation had a significant impact on bowel movement. The longer the time until the first day of nutrition/defecation, the later the start of proper bowel movement (OR, 1.23; *p* = 0.003; OR, 1.2; *p* = 0.005). However, it is unclear whether earlier enteral feeding led to an earlier bowel movement or if the patients did not start eating because of improper bowel functioning linked with vomiting, diarrhea, nausea, and paralysis.

All in all, loop ileostomy reversal is a safe operation with low rates of mortality (0.7%) and major complications, defined as CDC $\ge$ 3 (7.7%), but a relatively high rate of minor postoperative complications (69.3%), whereas merely the use of oral laxatives or prokinetic agents was considered a complication. Predictors leading to postoperative complications were a higher BMI, delayed time to bowel movement, and primary malignant disease, while stapled anastomoses seemed to be protective. Other independent predictors include ASA grade [30, 31], functional status, prolonged operative time, disseminated cancer, history of COPD, dialysis [32], delayed ileostomy reversal [33], and small bowel resection [6]. Furthermore, the two main risk factors for reoperation after loop ileostomy reversal were a higher BMI and anemia [12], while the level of training of the surgeon showed no association with immediate reoperation nor a higher rate of complications [6, 34, 35].

As it is everyone’s right to receive the best possible medical treatment, it is important to ensure good training facilities for the rising generation of doctors. Increasing surgical volume and years of practice are associated with improved performance [36], especially for the first years of learning. Therefore, it is common practice to slowly get residents involved in certain operative procedures by letting them perform increasingly more of the operation step-by-step until they can perform the whole operation. While the different extent of residents’ involvement in the loop ileostomy reversal in this retrospective study must be considered, it does not change the conclusion that it is not only safe but very important to teach residents surgical procedures early in order to enable their maturation into the new generation of skilled surgeons.

### Limitations

This study has some limitations. First, data from a small cohort in a retrospective database from a single center were used for the analyses. Second, limitations could also result from the chosen statistical methods. Only parameters that were statistically significant in the univariable analysis were included in the multivariable regression analysis. It is possible that insignificant variables in the univariable analysis became significant in the multivariable analysis using other methods of variable entry, such as forward or backward selection. Third, information on the exact level of resident participation is not included in the operation reports. As such, it is possible that residents only performed parts of the surgery, even when stated as first surgeons; however, it is also possible that they performed parts of the surgeries of attending surgeons.

## Conclusion

DLI reversal can be safely performed by surgical residents. Even with an increased surgery time, postoperative morbidity did not differ between the groups. The side-to-side stapled suture technique was utilized significantly more often by attending surgeons and was a statistically significant protective factor for intestinal motility dysfunction. Therefore, whether the benefit of receiving training in end-to-end hand-stitched sutures in a DLI reversal outweighs the improved intestinal motility of a side-to-side stapled suture warrants further consideration.

### Supplementary Information

Below is the link to the electronic supplementary material.

Supplementary file1 (DOCX 25 KB)

## Data Availability

The datasets generated and analyzed in the current study are not publicly available due to the Ethics Committee restrictions but are available from the corresponding author upon reasonable request.
