# Supplementary material for: Short-term outcome of diverting loop ileostomy reversals performed by residents: a retrospective cohort prognostic factor study
Source: Int J Colorectal Dis. 2023 Apr 21;38(1):108. doi: 10.1007/s00384-023-04390-0 (PMC10121496; doi:10.1007/s00384-023-04390-0)
Supplement: Supplementary file 1 — Supplementary file1 (DOCX 25 KB) [file 384_2023_4390_MOESM1_ESM.docx]

|  | **Complications Grade** $\boldsymbol{\geq3}$ | | | |
| --- | --- | --- | --- | --- |
|  | **OR** | **95% CI** | **B coefficient (df)** | **p-value** |
| Sex  Male | 1.461 | 0.582-3.667 | 0.379 (1) | 0.420 |
| Age | 0.998 | 0.969-1.027 | -0.002 (1) | 0.877 |
| BMI (kg/m^2^) | 0.953 | 0.863-1.052 | -0.048 (1) | 0.338 |
| ASA group  ASA $\geq$3 | 0.313 | 0.077-1.275 | -1.162 (1) | 0.105 |
| Comorbidities  Cardiovascular system  Diabetes mellitus | 0.870  0.839 | 0.369-2.050  0.239-2.950 | -0.139 (1)  -0.175 (1) | 0.750  0.785 |
| Primary disease  malignant | 1.341 | 0.511-3.518 | 0.294 (1) | 0.551 |
| Duration of surgery (min) | 0.986 | 0.969-1.004 | -0.014 (1) | 0.132 |
| Anastomotic type  Side-to-side stapled suture | 1.283 | 0.359-4.581 | 0.249 (1) | 0.701 |
| Wound closure technique  PSC | 1.304 | 0.497-3.422 | 0.266 (1) | 0.589 |
| Level of training  Surgical trainee | 0.520 | 0.199-1.360 | -0.654 (1) | 0.182 |
| Time to reversal (months) | 1.002 | 0.999-1.004 | 0.002 (1) | 0.200 |
| Delay time to reversal  $\geq$120 days | 1.628 | 0.690-3.838 | 0.487 (1) | 0.266 |
| Bowel movement  > three days after OP | 4.348 | 1.670-11.321 | 1.470 (1) | **0.003** |

Supplementary 1 Univariable logistic regression analysis of risk factors for CDC [3] complications grade $\boldsymbol{\geq}$ **3.**

|  | **Surgical site infections** | | | | | | | |
| --- | --- | --- | --- | --- | --- | --- | --- | --- |
|  | **Univariable Regression** | | | | **Multivariable Regression** | | | |
|  | **OR** | **95% CI** | **B coefficient (df)** | **p-value** | **OR** | **95% CI** | **B coefficient (df)** | **p-value** |
| Sex  Male | 1.486 | 0.585-3.775 | 0.396 (1) | 0.405 |  |  |  |  |
| Age | 1.012 | 0.978-1.047 | 0.012 (1) | 0.497 |  |  |  |  |
| BMI (kg/m^2^) | 1.096 | 1.005-1.195 | 0.092 (1) | **0.037** | 1.162 | 1.043-1.294 | 0.150 (1) | **0.007** |
| Comorbidities  Cardiovascular system  Diabetes mellitus | 2.049  0651 | 0.784-5.358  0.145-2.920 | 0.717 (1)  -0.429 (1) | 0.144  0.575 |  |  |  |  |
| Primary disease  malignant | 1.004 | 0.370-2.729 | 0.004 (1) | 0.993 |  |  |  |  |
| Duration of surgery (min) | 1.008 | 0.995-1.022 | 0.008 (1) | 0.231 |  |  |  |  |
| Anastomotic type  Side-to-side Stapled suture | 0 | 0 | -18.630 (1) | 0.998 |  |  |  |  |
| Wound closure technique  PSC | 0.760 | 0.289-1.997 | -0.275 | 0.578 |  |  |  |  |
| Level of training  Surgical trainee | 1.420 | 0.559-3.607 | 0.351 (1) | 0.461 |  |  |  |  |
| Time to reversal (months) | 0.998 | 0.993-1.003 | -0.002 (1) | 0.388 |  |  |  |  |
| Delay time to reversal  $\geq$120 days | 0.869 | 0.339-2.227 | -0.140 (1) | 0.770 |  |  |  |  |
| Bowel movement  > three days after surgery | 3.250 | 1.123-9.402 | 1.179 (1) | **0.030** | 3.973 | 1.300-12.138 | 1.379 (1) | **0.015** |

Supplementary 2 **Univariable and multivariable logistic regression analysis for risk factors for surgical site infections.**

|  | **Intestinal motility dysfunction** | | | | | | | |
| --- | --- | --- | --- | --- | --- | --- | --- | --- |
|  | **Univariable logistic regression** | | | | **Multivariable logistic regression** | | | |
|  | **OR** | **95% CI** | **B coefficient (df)** | **p-value** | **OR** | **95% CI** | **B coefficient (df)** | **p-value** |
| Sex  Male | 1.398 | 0.816-2.394 | 0.335 (1) | 0.222 |  |  |  |  |
| Age | 1.010 | 0.992-1.028 | 0.009 (1) | 0.301 |  |  |  |  |
| BMI (kg/m^2^) | 0.975 | 0.920-1.034 | -0.025 (1) | 0.401 |  |  |  |  |
| ASA Group  ASA $\geq$3 | 1.057 | 0.500-2.232 | 0.055 (1) | 0.885 |  |  |  |  |
| Comorbidities  Cardiovascular system  Diabetes mellitus | 1.115  1.151 | 0.654-1.902  0.530-2.501 | 0.109 (1)  0.141 (1) | 0.688  0.723 |  |  |  |  |
| Primary disease  malignant | 1.801 | 1.033-3.141 | 0.589 (1) | **0.038** | 1.980 | 1.120-3.500 | 0.683 (1) | **0.019** |
| Duration of surgery | 0.994 | 0.985-1.003 | -0.006 (1) | 0.196 |  |  |  |  |
| Anastomotic type  Side-to-side Stapled suture | 0.378 | 0.176-0.809 | -0.973 (1) | **0.012** | 0.337 | 0.155-0.733 | -1.088 (1) | **0.006** |
| Wound closure  PSC | 0.501 | 0.250-1.003 | -0.691 (1) | 0.051 |  |  |  |  |
| Level of training  Surgical trainee | 0.625 | 0.366-1.068 | -0.469 (1) | 0.086 |  |  |  |  |
| Time to reversal | 1.000 | 0.998-1.001 | 0.000 (1) | 0.643 |  |  |  |  |
| Delay time to reversal  $\geq$120 days | 0.962 | 0.565-1.638 | -0.038 (1) | 0.887 |  |  |  |  |

Supplementary 3 **Univariable and multivariable logistic regression analysis for risk factors for intestinal motility dysfunction**
